# Supplementary material for: Using natural language processing to identify acute care patients who lack advance directives, decisional capacity, and surrogate decision makers
Source: PLoS One. 2022 Jul 11;17(7):e0270220. doi: 10.1371/journal.pone.0270220 (PMC9273092; doi:10.1371/journal.pone.0270220)
Supplement: Attachment — Submitted filename: INEADS response to reviewers.docx [file pone.0270220.s002.docx]

Giovanni Ottoboni, Psy, PhD

Academic Editor, PLOS ONE

May 2, 2022

RE: PONE-D-22-04209

Dear Dr. Ottoboni,

Thank you for the opportunity to revise and resubmit our manuscript titled “Using Natural Language Processing to Identify Acute Care Patients Who Lack Advance Directives, Decisional Capacity, and Surrogate Decision Makers.” We have enclosed a revised manuscript and provided responses to the reviewers below. We declare no potential conflicts of interest. This study was funded by the National Institute of Nursing Research, National Institutes of Health (R21 NR019319).

Thank you for your time and consideration.

Sincerely,

Bevin Cohen

| Editorial Office | |
| --- | --- |
| 1. Please ensure that your manuscript meets PLOS ONE's style requirements, including those for file naming. | Thank you, we have made revisions according to the author guidelines. |
| 2. Please ensure that you include a title page within your main document; we do require this to be part of the manuscript file itself and not uploaded separately. | We included the title page in the main document. |
| 3. Thank you for stating the following in the Acknowledgments Section of your manuscript:  "This study was funded by National Institute of Nursing Research [NINR] (R21 NR019319), “Improving ethical care for patients who are incapacitated with no evident advance directives or surrogates (INEADS)”. The content is solely the responsibility of the authors and does not necessarily represent the official views of the National Institute of Nursing Research. The funders had no role in study design, data collection and analysis, decision to publish, or preparation of the manuscript."  We note that you have provided funding information that is not currently declared in your Funding Statement. However, funding information should not appear in the Acknowledgments section or other areas of your manuscript. We will only publish funding information present in the Funding Statement section of the online submission form.  Please remove any funding-related text from the manuscript and let us know how you would like to update your Funding Statement. Currently, your Funding Statement reads as follows:  "Our study was funded by National Institute of Nursing Research [NINR] (R21 NR019319), “Improving ethical care for patients who are incapacitated with no evident advance directives or surrogates (INEADS)”. The funders had no role in study design, data collection and analysis, decision to publish, or preparation of the manuscript."  Please include your amended statements within your cover letter; we will change the online submission form on your behalf. | We removed the funding statement from the manuscript. Our funding information is confirmed below.  This study was funded by the National Institute of Nursing Research, National Institutes of Health (R21 NR019319). |
| 4. PLOS requires an ORCID iD for the corresponding author in Editorial Manager on papers submitted after December 6th, 2016. Please ensure that you have an ORCID iD and that it is validated in Editorial Manager. | We have linked the corresponding author’s ORCID ID. |
| 5. Please amend either the abstract on the online submission form (via Edit Submission) or the abstract in the manuscript so that they are identical. | Thank you, we made edits to the abstract and updated in the manuscript and system. |
| 6. Your ethics statement should only appear in the Methods section of your manuscript. If your ethics statement is written in any section besides the Methods, please move it to the Methods section and delete it from any other section. Please ensure that your ethics statement is included in your manuscript, as the ethics statement entered into the online submission form will not be published alongside your manuscript. | We have removed the ethics statement from the acknowledgements section. It now appears only in the methods section. |
| Reviewer 1 | |
| 7. Study dataset and population: could the authors please provide more information regarding the hospital in which the data is collected (e.g. university or general hospital etc.)? | We have expanded the description of the hospital. |
| 8. If patients for instance completed an advance directive with the general practitioner or another primary care provider, might there be a chance that this is included in the clinical hospital notes? Are there shared electronic patient files available between different settings? | Thank you for raising this comment. Given that these data come from a de-identified dataset, it was not possible for us to review files that that were entered into the electronic health record as scanned documents. However, generally speaking, the issue you raise is one that continues to pose challenges across care settings and health systems. Sometimes, if a primary care provider is in the same health system as a hospital, it may be possible for the documents to appear automatically on the inpatient record if a patient is admitted. However, not all electronic health records systems operate this way. Moreover, when patients’ primary care providers are not affiliated with the hospital to which they are admitted, or they use a different electronic health record, transfer of advance directives is unlikely to be automated. The new eMOLST program attempts to solve this issue by creating a central repository of MOLST forms that can be accessed across platforms and institutions, but this was not in place at the time the study data were collected, and at present the use of eMOLST is limited in many areas. |
| 9. The authors report that 45% of patients died within 4 years after discharge, did the authors collect any information regarding the setting where patients died? Was this data collected in the clinical notes of the hospital? Was this data also collected for those who died outside the hospital? This might need some clarification in the methods section. | We appreciate the point you raise in this comment and wish we were able to clarify further. However, these data are from a publically available database that does not provide more detailed information. |
| 10. The authors found that only a small percentage of patients were characterized as INEADS, could the authors please provide explanations for this low percentage? Might there be an underestimation of this phenomenon? | Thank you for raising this. We added a comment about this in the limitations section. |
| 11. Could the authors please provide more information on whether this algorithm can be used in other hospitals and healthcare contexts? Should the algorithm be validated again prior using it in for instance another hospital? | Thank you for raising this. Indeed our hope is that this algorithm would have broad applicability across health systems—particularly those with similar characteristics serving similar patient populations. However, we agree that the algorithm would need to be validated for use in those contexts and may require some modification to reflect local documentation practices and patient populations. We have added to our discussion of this in the limitations section. |
| Reviewer 2 | |
| 12. The authors should justify the reason why other methods were not tested and only NimbleMiner was used, or at least present a more comprehensive state-of-the-art regarding NLP and electronic health records | Thank you for this suggestion. We have provided a justification for this choice in the methods section. |
| 13. The authors stated that their findings underline the importance of encouraging patients to complete advance directives, particularly when they lack social support. This is not that straightforward from the results, although it can be deduced by comparing the high likelihood of being INEADS demographic with the full cohort. However, this does not seem to be the core of the study, based on what the authors stated (purpose of the study: develop and validate an NLP algorithm to identify patients who are INEADS using documentation …). I would suggest the authors clarify the purpose of the study (develop and validate an NLP algorithm, or investigate the characteristics of INEADS patients?) and be consistent with the conclusions. | Thank you. We have amended the conclusion in the abstract and main manuscript. |
| 14. The validation has been made on a very small dataset (10% sample, corresponding to 8 patients), and the authors stated that those patients “appeared to meet the definition of INEADS”, but it is not possible to be completely sure about it, since this information was not reported in the original dataset. | This is correct; INEADS status is not a universally defined construct and therefore is not documented in any medical records. Our ‘gold standard’ assessment was based on expert review of the full set of clinical notes for each admission. |
| 15. Authors stated that “the purpose of the study was to develop and validate a natural language processing (NLP) algorithm to identify patients who are INEADS using documentation in electronic clinical notes”. This is partially true: the applied NLP method is meant to find words belonging to specific categories whose presence is likely to identify INEADS patients. Authors use an arbitrary threshold for the number of the identified categories and, based on the number of identified categories, patients are labeled with a high or low risk of being INEADS. | Thank you for raising this. We acknowledge that this is an exploratory and descriptive study, intended as an initial attempt to identify patients who may be INEADS. As this is the first study of its kind and one of the first studies to characterize this patient population, there is no previous work to build from with regard to which categories or how many categories suggest that a person might be INEADS. Summarizing and reporting the data required us to make some decisions about how to categorize and characterize patients. Since there is no previously validated method for doing so, we relied on extensive discussion and consensus among our interdisciplinary team, which included physician, nurse, social work, public health, data science, and bioethics representation. |
| 16. Key findings are well reported, but no literature regarding the same topic (i.e., NLP and INEADS patients) was provided. Later in the manuscript, the authors stated that this is the first study attempting to develop NLP algorithms to detect INEADS patients. However, the introduction should include more information about the application of NLP using electronic health records to make the reader better understand what NLP is meant to do. Please add this information in the abstract and the introduction and revise accordingly. | We have revised the introduction per this suggestion. |
| 17. I would suggest adding a different graphical representation for the t-test and chi-square test for clarity. | It does not appear common to make this graphical distinction, but we would be happy to make this change if the journal prefers and it conforms to their editorial standards. To help with clarity, we added specification in the table footnote. |
| 18. I would not use “NLP methods” as the name of the subsection. Instead, I would use “NLP algorithm pipeline”. The word “methods” can be misleading since it seems that more than one method is developed when only one is presented. | We have made this change. |
| 19. Page 10, line 15: “We then created subcategories within each domain”. How were the subcategories created? No information is given. Please, explain in a more extensive way revise accordingly. | We have clarified and provided additional detail about this process. |
| 20. Page 10, line 16: “The 17 subcategories were grouped based on whether they indicated reduced or elevated potential for being INEADS”. Instead of “grouped”, I would suggest to the authors to use “annotated” or something similar, since “reduced” or “elevated” is a characteristic of that subgroup. Also, please change “they indicated reduced…” to “they indicated the reduced …”. | We have made these changes. |
| 21. Page 11, line 1: “Four team members (JS, BC, AL, MT) developed the initial list of terms, which was iteratively revised and finalized by all team members. Table 1 lists examples of terms in each category”. I would suggest giving the full list of terms within each category as supplementary materials for reproducibility purposes, since only a few examples are available in the main text. | We have included the full list in a supplemental file and added a reference to this material in the manuscript. |
| 22. Page 12, line 3: “To evaluate NLP algorithm performance, we randomly sampled a gold standard testing set of 300 clinical notes from all clinical notes of patients with diagnoses that could be associated with being INEADS”. How has the gold standard testing been made? The 300 clinical notes were chosen randomly, or did the authors use a specific rule? Please add information about the note selection in the manuscript. | We revised the order of the text to clarify that the notes were randomly selected from a cohort of patients who have diagnoses that may put them at higher risk of being INEADS. |
| 23. NLP algorithm performances were evaluated in detecting the 17 subcategories, not in detecting a potential INEADS patient. For this reason, maybe the main research question should be rephrased (INEADS patients are detected based on the arbitrary number (4) of identified categories with an elevated potential of being INEADS). | Thank you, we have revised the research question to be more precise. |
| 24. Page 12, line 23: “…, we identified admissions that included at least one of the five subcategories indicating elevated potential for being INEADS”. The subcategories indicating the elevated potential of being INEADS presented in Table 1 are 6. The “lacking capacity” subcategory is missing in this part of the methods. Only 5 subcategories indicating elevated potential of being INEADS are mentioned throughout the papers. Change it accordingly to what is reported in Table 1. | Thank you for identifying this typographical oversight. The sentence should specify “social” subcategories, as it appears elsewhere in the manuscript and table. We have made this change. |
| 25. Page 13, line 2: “surrogate decision maker unknown”. Be consisted of what is written in Table 1, “surrogate decision maker unidentified”. | Thank you for catching this error. We revised throughout the manuscript. |
| 26. Page 13, line 4: “We validated the “high likelihood of being INEADS” classification by conducting a case study validation on 10% of this cohort”. Here the 10% means 8 hospital admissions. It is a very small number for the results to be significant. I would suggest validating the classification on a higher number of hospital admissions. | Thank you for this suggestion. To provide a better validation process, we bifurcated the validation cohort further to distinguish patients who had no ‘protective’ factors from those who had at least one. We doubled the number of charts reviewed in the validation by reviewing eight in each group. |
| 27. Page 13, line 8: “Lastly, we compared demographic characteristics of the “possibility of being INEADS” cohort and the “high likelihood of being INEADS” cohort against the full cohort using t-tests or chi-square tests”. For the sake of clarity, I suggest to the authors to add specifications on which kind of data (i.e., continuous variables or categorical data) these two statistical methods are applied. | We have added this. |
| 28. Page 13, line 17: “The most common type of insurance was Medicare”. For a non-American audience, it may be helpful to better explain what “Medicare and Medicaid” stand for (the Medicaid is mentioned in the discussion section). | Thanks for this suggestion. We have added an explanation. |
| 29. Page 13, line 21: I would change “NLP algorithm performance against gold standard” in “Validation of NLP algorithm on gold standard testing set”. | We have made this change. |
| 30. Page 14, line 2: “Consistent with the proportion of each type of note in the dataset overall, 68% of notes containing at least one subcategory were nursing notes and 18% were physician notes”. What about the remaining 14%? Which is the source of the remaining notes? | We clarified in the methods that the remaining note types collectively comprised only 7% of all notes, and in the results that these note types collectively comprised 14% of notes containing any subcategory. |
| 31. Page 14, line 4: “However, subcategories indicating elevated potential for being INEADS were most likely to be documented in consult notes (57%) and least likely to be documented in nursing notes (48%)”. Is “consult notes” a synonym of “physician notes”? If yes, I suggest remaining consistent using the same terminology throughout the paper. | Thank you for the opportunity to clarify. Consult notes are not synonymous with physician notes; they are coded as a separate category. Given that the dataset is publically available, we do not have additional information on which providers wrote consult notes. However, generally speaking, such notes typically reflect the involvement of a specialty service called for challenging cases, for example, an ethics or palliative care team. |
| 32. Page 14, line 17: “Less than one percent of admission (n=81) met the criteria for the “high likelihood of being INEADS” cohort, with 79 containing four and two containing all five of the subcategories”. Are the subcategories indicating elevated potentials of being INEADS six? | Please see clarification above. This classification included the five social subcategories only. |
| 33. Page 16, line 21: “However, with more adults living in social isolation”. Please add a reference for this sentence. | Thank you, we have added a reference. |
| 34. Page 17, line 15: “Second, the study is limited by its relatively small number of annotated notes used for training and testing”. Is it right to use “training” for this type of application? The method has pre-defined rules (words within subcategories) and is manually enhanced by researchers by adding some synonyms, so the algorithm does not seem to be “trained” following the strict definition used in machine learning field (https://docs.aws.amazon.com/machine-learning/latest/dg/training-ml-models.html). The algorithm does not seem to “learn” something from the data, rather the authors set rules to identify words. | Thanks for this comment. We agree and have revised the language. |
| 35. Page 18, line 10: “Our results underscore the importance of encouraging patients to complete advance directives, particularly when they lack social support”. This conclusion is not so straightforward, based on the implemented methods. Authors should try to justify better their conclusions based on their results. | Thank you, we have revised the conclusion. |
